# Dazomet fumigation modification of the soil microorganism community and promotion of *Panax notoginseng* growth

**DOI:** 10.3389/fmicb.2024.1443526

**Published:** 2024-07-26

**Authors:** Ya-meng Lin, Ming-hua Li, Chun-yan Dai, Yuan Liu, Wen-ping Zhang, Qian Yang, Xiu-ming Cui, Ye Yang

**Affiliations:** ^1^Faculty of Life Science and Technology, Kunming University of Science and Technology, Kunming, China; ^2^Key Laboratory of Panax notoginseng Resources Sustainable Development and Utilization of State Administration of Traditional Chinese Medicine, Kunming, China; ^3^Yunnan Provincial Key Laboratory of Panax notoginseng, Kunming, China

**Keywords:** Dazomet, fumigation, *Panax notoginseng*, saponins, soil microbial diversity, function

## Abstract

**Introduction:**

*Panax notoginseng,* a medicinal herb in China, is attacked by several pathogens during its cultivation. Dazomet (DZ) is a soil fumigant that is effective in controlling soil-borne pathogens, but its long-term effects on *P. notoginseng* growth and soil properties are unknown.

**Methods:**

We conducted field experiments over two consecutive years to assess the impact of three concentrations of DZ fumigation (35 kg/666.7 m^2^, 40 kg/666.7 m^2^, and 45 kg/666.7 m^2^) on soil physicochemical properties, microbial diversity, and *P. notoginseng* growth. Correlation analyses were performed between microbial community changes and soil properties, and functional predictions for soil microorganisms were conducted.

**Results:**

DZ fumigation increased total nitrogen, total phosphorus, total potassium, available phosphorus, available potassium, and ammonia nitrogen levels in the soil. DZ fumigation promoted the nutrient accumulation and improvement of agronomic traits of *P. notoginseng*, resulted in a 2.83–3.81X yield increase, with the highest total saponin content increasing by 24.06%. And the 40 kg/666.7 m^2^ treatment had the most favorable impact on *P. notoginseng* growth and saponin accumulation. After DZ fumigation, there was a decrease in the relative abundance of pathogenic fungi such as *Fusarium*, *Plectosphaerella*, and *Ilyonectria*, while beneficial bacteria such as *Ramlibacter*, *Burkholderia*, and *Rhodanobacteria* increased. The effects of fumigation on soil microorganisms and soil physicochemical properties persisted for 18 months post-fumigation. DZ fumigation enhanced the relative abundance of bacteria involved in the biosynthesis of secondary metabolites and arbuscular mycorrhizal fungi, reduced the relative abundance of plant–animal pathogenic fungi, reduced the occurrence of soil-borne diseases.

**Conclusion:**

In conclusion, DZ fumigation enhanced soil physicochemical properties, increased the proportion of beneficial bacteria in the soil, and rebalanced soil microorganism populations, consequently improving the growth environment of *P. notoginseng* and enhancing its growth, yield, and quality. This study offers a theoretical foundation for DZ fumigation as a potential solution to the continuous cropping issue in perennial medicinal plants such as *P. notoginseng*.

## 1 Introduction

*Panax notoginseng* (Araliaceae), a traditional Chinese medicinal herb used for blood-stasis dispersal, bleeding cessation, and reduction of pain and swelling, is commonly used to prevent and treat traumatic injuries, cardiovascular, and cerebrovascular diseases. The primary active components of *P. notoginseng* include saponins, polysaccharides, amino acids, and volatile oils (Liu et al., 2020). Saponins exhibit activities such as anti-inflammatory, anticoagulant, and anti-tumor effects (Cheng et al., 2024). Polysaccharides from *P. notoginseng* have pharmacological properties that enhance immunity, lower blood sugar, mitigate radiation effects, reduce inflammation, and provide antioxidant benefits (Xing et al., 2021). Dencichines, found in the amino acids of *P. notoginseng*, has been identified as the key active ingredient for its hemostatic effect (Ji et al., 2023). In 2022, *P. notoginseng* in China was grown on 24,300 hectares and generated a total value of approximately 5.6 billion US dollars. However, continuous cropping poses significant challenges for successful *P. notoginseng* cultivation. For example, planting at intervals of less than 10 years can result in greatly reduced yields. During continuous growth of *P. notoginseng*, the deterioration of soil physicochemical properties and the accumulation of allelochemicals can cause an imbalance in the rhizosphere microbial community. This can result in increased pathogen populations causing *P. notoginseng* disease outbreaks (Ling et al., 2014; Tan et al., 2017).

Soil fumigation is an effective strategy employed to manage soil-borne diseases and address continuous cropping challenges. Dazomet (DZ) is commonly used due to its efficacy and low toxicity. DZ fumigation can rebalance the soil microbial ecosystem by reducing soil pathogens and enhancing populations of beneficial bacteria. Following DZ soil fumigation in apple orchards, biocontrol bacteria like *Pseudomonas, Bacillus,* and *Sphingomonas* increased while fungal pathogens such as *Fusarium*, *Pseudollescheria*, and *Kernia* declined. These changes fostered apple seedling growth (Chen et al., 2022). Dazomet is used to prevent soil-borne diseases in fruits, vegetables and Chinese herbal medicinal material, like rape clubroot, watermelon phytophthora blight and cucumber fusarium wilt (Tian et al., 2014; Hwang et al., 2017; Ge et al., 2021). At an application rate of 274.5 kg/hm^2^, DZ prevented chrysanthemum rotavirus damage and wilt disease occurrence (Castello et al., 2022). DZ also reduced American ginseng root rot disease incidence by 69.39% (Peng et al., 2024), inhibited ginger root knot nematodes for up to 12 weeks, and improved ginger yields by 37.37% (Eo and Park, 2014; Wang et al., 2022).

DZ fumigation controls soil-borne diseases and also enhances crop agronomic traits. For example, DZ fumigation increased strawberry plant height, chlorophyll content and fresh weight. This increased soluble solids in fruits by 4.40% and yield by 4.32% (Li et al., 2023), in addition to improving the survival of *Codonopsis pilosula* (Campanulaceae) by 42.4% (Wang et al., 2021); the abundance of morel mycelium was also enhanced, and the number and yield of morel fruiting bodies increased (Chen et al., 2023).

To reduce the continuous cropping challenges of *P. notoginseng* cultivation, we studied the effects of DZ fumigation on soil physicochemical properties, microbiota and *P. notoginseng* growth over two consecutive years. The goal of the present study was to determine optimal DZ fumigation methods for reducing disease problems, particularly for perennial medicinal plants such as *P. notoginseng*.

## 2 Materials and methods

The experiment was conducted in Bozhu Town, Wenshan Zhuang and Miao Autonomous Prefecture, Yunnan Province (103°55′19″E, 23°27′18″N). Our experiment started in 2020 and continued until it concluded in 2022. The study utilized red soil that had been cultivated with *P. notoginseng* since 2010. One-year-old *P. notoginseng* seedlings were used (Yunnna Qidan Pharmaceutical Co., Ltd.).

### 2.1 Fumigation experiment

The field trial plots were 2 × 11 m, incorporating four concentration treatments with three replicates: CK (0 kg/666.7 m^2^), DZ35 (35 kg/666.7 m^2^), DZ40 (40 kg/666.7 m^2^), and DZ45 (45 kg/666.7 m^2^). Fumigation commenced on November 2, 2020. After plowing the plots to a depth of 20–30 cm, dazomet (DZ, C_5_H_10_N_2_S_2_, Nantong Shizhuang Chemical Co., Ltd.) was evenly applied to the soil surface, thoroughly mixed using a rotary tiller and immediately covered with 0.06 mm thick plastic film (Shandong Longxing Technology Co., Ltd.). The film was removed on December 7, 2020, after 35 days of fumigation, and the *P. notoginseng* seedlings were transplanted on December 27, 2020, following local agricultural practices.

### 2.2 Soil and plant sample collection

Soil samples were collected on December 7, 2020 (1 month post-fumigation) and March 26, 2022 (18 months post-fumigation) at a depth of 0–15 cm using a five-point sampling method (Bao, 2000). Samples were labeled CK1, DZ351, DZ401, and DZ451 for the 1 month post-fumigation, and CK2, DZ352, DZ402, and DZ452 for the 18 month post-fumigation. Each sample was divided into two parts: one part was sieved through 2 mm mesh, frozen in liquid nitrogen, and stored at −80°C for microbial analysis. The other part was air-dried naturally for analysis of physicochemical properties.

Samples of *P. notoginseng* plants were collected on September 29, 2021, from each treatment, including 15 randomly selected *P. notoginseng* plants. Parameters such as leaf area, length, width, thickness, and weights of different parts of *P. notoginseng* were measured using appropriate tools. These samples were dried, powdered, sieved, and stored for nutrient element analysis.

### 2.3 Physicochemical properties of soil

Soil N, P and K contents were measured as described in Soil and Agricultural Chemistry Analysis (Bao, 2000). In brief, total nitrogen (TN) was determined by the semi-micro Kjeldahl method (ZG/KDN-102F). Ammonium nitrogen (NH_4_^+^-N) was determined by the 2 mol·L^−1^ KCl extraction - indophenol blue spectrophotometry (UV-2600, 625 nm). Nitrate nitrogen (NO_3_^−^-N) was determined by the CaSO_4_ and H_2_O extraction - Phenol disulfonic acid colorimetric method (UV-2600, 420 nm). Total phosphorus (TP) was determined by HClO_4_-H_2_SO_4_ digestion - molybdenum antimony colorimetric method (UV-2600, 880 nm). Available phosphorus (AP) was determined by the 0.5 mol·L^−1^ NaHCO_3_ extraction - molybdenum antimony colorimetric method (UV-2600, 880 nm). Total potassium (TK) was determined by the NaOH fusion - flame photometric method (FP6410). Available potassium (AK) was determined by the 1.0 mol·L^−1^ NH_4_OAc extraction-flame photometric method (FP6410).

### 2.4 Nutrient element determination in plants

Analysis of total nitrogen (N), total phosphorus (P), and total potassium (K) content in plants used the NY/T1017-2011 standard. The plant samples were digested with H_2_SO_4_-H_2_O_2_, and N was determined using the Kjeldahl method (ZG/KDN-102F), P content was determined molybdenum antimony colorimetric method (UV-2600, 880 nm), and K content was determined using flame photometry (FP6410).

### 2.5 Saponin content determination

The sample solutions were prepared according to the method described in Chinese Pharmacopoeia (2020). A total of 0.3 g of each sample was weighed, 10 ml of 70% methanol was added, and the mixture was extracted by ultrasonication for 30 min and centrifuged at 3,000 × g for 15 min. Then, the supernatant was harvested, the residues were extracted again, and the supernatants of the two extracts were combined. Next, 25 ml of 70% methanol was added, and the sample solution was filtered through a 0.22-μm filter membrane.

HPLC equipment (Agilent 1,200) was equipped with a column (BDS C18, 250 × 4.6 mm, 5 μm) and a detector (203 nm) packed with ODS-bonded silica gel (5 μm particle size). The separation system consisted of water (A) and acetonitrile (B). The injection volume was 10 μl, and the flow rate was 1.0 ml/min. T The chromatographic conditions included elution for 0–25 min with 20% B, 25–30 min with 25% B, 30–41 min with 41% B, 41–55 min with 45% B, 55–80 min with 74% B, 80–90 min with 100% B and 90–100 min with 20% B. The concentrations of saponins were obtained according to the standard curve and peak area.

### 2.6 High-throughput sequencing of soil microbiota

The DNA was extracted with the TGuide S96 Magnetic Soil/Stool DNA Kit (Tiangen Biotech (Beijing) Co., Ltd., DP812). The universal primers for 16S are F (AGRGTTTATYNTGGCTCAG), R (TASGGHTACCTGTTASGAGATT), and the universal primers for ITS are F (CTTGGTCATTTAGAGGAAGTAA) and R (TCCTCCGCTTATTGATGATTGC). A quality-controlled library is sequenced using PacBio Sequel. The raw subreads are corrected to obtain Circular Consensus Sequencing (CCS) sequences using SMRT Link version 8.0. Subsequently, the lima software (v1.7.0) is employed to identify CCS sequences of different samples based on barcode sequences. The cutadapt 1.9.1 software is used for primer sequence identification and removal, and length filtering to obtain Clean-CCS sequences devoid of primer sequences. The UCHIME v4.2 software is then utilized to identify and remove chimeric sequences, resulting in Effective-CCS sequences.

### 2.7 Data processing and analysis

OTU clustering: The sequences are clustered at a 97% similarity level using USEARCH version 10.0 (Edgar, 2013), with a default threshold filter of 0.005% of the total sequenced reads for OTUs (Bokulich et al., 2013). Species annotation: Using the SILVA (16S) and UNITE (ITS) reference databases, the feature sequences are taxonomically annotated using a naive Bayesian classifier combined with alignment methods. This approach provides species classification information for each feature, with a classifier confidence level of 0.7. Subsequently, the community composition of each sample is analyzed at various taxonomic levels (phylum, class, order, family, genus, species) using the QIIME software to generate species abundance tables. Finally, R language tools are utilized to visualize the community structure of each sample at different taxonomic levels. Functional predictions for bacteria and phenotypic predictions for fungi are, respectively, performed using PICRUSt2 (16S) and FUNGuild (ITS). The bioinformatics analysis of this study was performed with the BMK online platform.^1^ The data is processed using Microsoft Excel 2021, and graphs are created using GraphPad Prism 9.5 and Origin 2018. Statistical analysis such as one-way ANOVA is conducted using SPSS 23 software.

## 3 Results

### 3.1 Effects of DZ fumigation on the growth and development of *Panax notoginseng*

**Figure 1:**
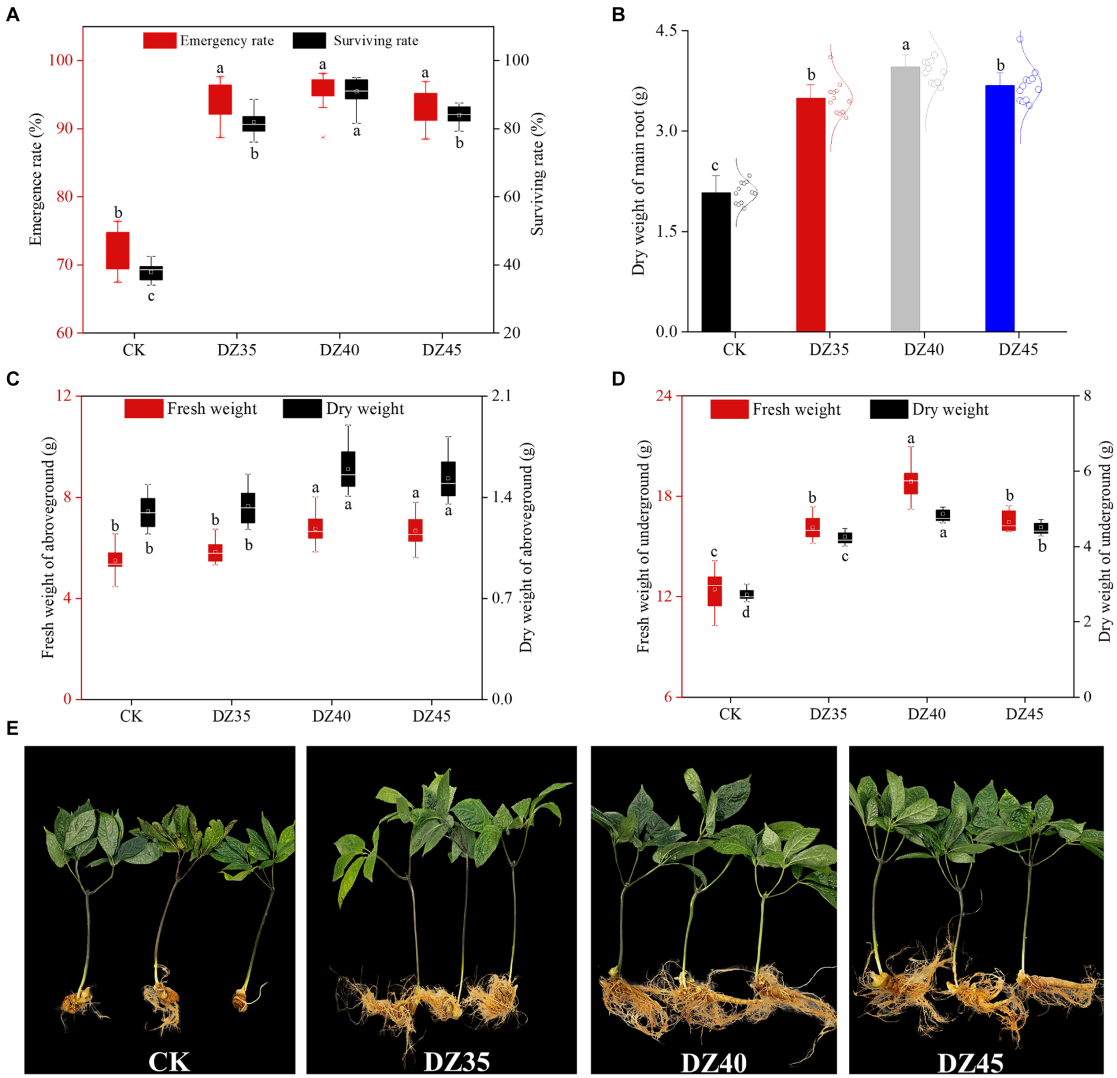
Effects of different concentrations of dazomet fumigation on emergence rate and survival rate **(A)**, main root weight **(B)**, aboveground weight **(C)**, underground weight **(D)** and plant growth **(E)** of *P. notoginseng*. Lowercase letters indicate significance at the *p* < 0.05 level (*n* = 15).

DZ fumigation significantly enhanced the emergence and survival rates of 2-year-old *P. notoginseng* (Figure 1A). Compared to CK, the emergence rates increased by 30.17–33.32% with DZ fumigation, and there were no significant differences among the DZ concentrations. The survival rates were 35.71% for CK and 81.6, 90.4, and 84% for the DZ35, DZ40, and DZ45 treatments, respectively. This indicated that DZ treatment greatly enhanced the emergence rate and survival rate of 2-year-old *P. notoginseng*, and the DZ40 treatment provided the most favorable outcome.

DZ fumigation promotes the growth of *P. notoginseng* plants, but there was no significant difference between different concentrations of dazomet (Supplementary Table 1; Supplementary Figure 1). The DZ treatment stimulated the accumulation of both aboveground and underground biomass of *P. notoginseng*. Compared to CK, the dry weight of the main root increased by 67.78, 90.06, and 76.83% under DZ35, DZ40, and DZ45 treatments, respectively (Figure 1B) and resulted in significant yield increases per acre of 2.83, 3.81 and 3.16X. In the DZ40 treatment, the aboveground dry weight of *P. notoginseng* was 19.32 and 4.247% higher than the DZ35 and DZ45 treatments, respectively, while the underground dry weight increased by 14.32 and 7.73%, respectively (Figures 1C,D). These results indicated that DZ fumigation enhanced *P. notoginseng* yield, with the largest effect produced by the DZ40 treatment.

### 3.2 Effects of DZ fumigation on the main saponins content of the main root

**Figure 2:**
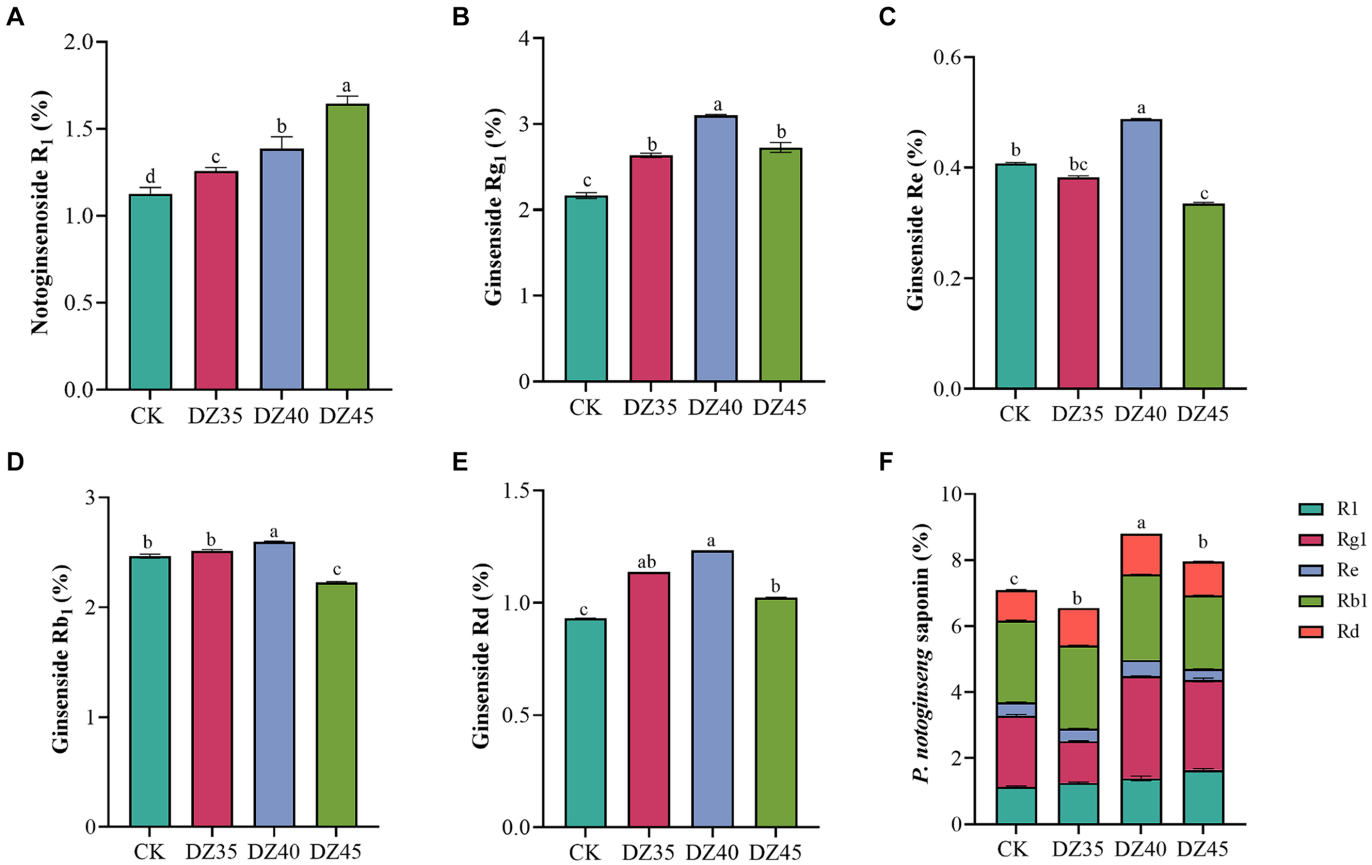
Effects of dazomet fumigation on the main saponins contents of *P. notoginseng*. Lowercase letters indicate significance at *p* < 0.05 level (*n* = 3).

DZ fumigation can alter the main saponins contents in the main roots of *P. notoginseng.* Compared to the CK, the content of R_1_, Rg_1_, and Rd. in the main roots increased by 11.63, 21.71, and 22.26%, respectively, with DZ35 treatment; by 23.8, 43.18, and 32.25%, respectively, with DZ40 treatment; and by 46, 25.96, and 10%, respectively, with DZ45 treatment. The content of Re and Rb_1_ changed by −5.9, 19.9, and − 17.44% and 2.27, 5.31, and − 9.57% (Figure 2). Under DZ40 and DZ45 treatments, the total saponin content (PNS, sum of R1, Rg1, Rd., Re, and Rb1) in the main roots was 8.80 and 7.96%, respectively, which were 24.06 and 12.15% higher than CK (Figure 2F). These results indicated that the PNS content increased after DZ fumigation, and DZ40 treatment showed the most significant promotion.

### 3.3 Effects of DZ fumigation on the levels of nitrogen, phosphorus, and potassium nutrients in plant and soil

**Figure 3:**
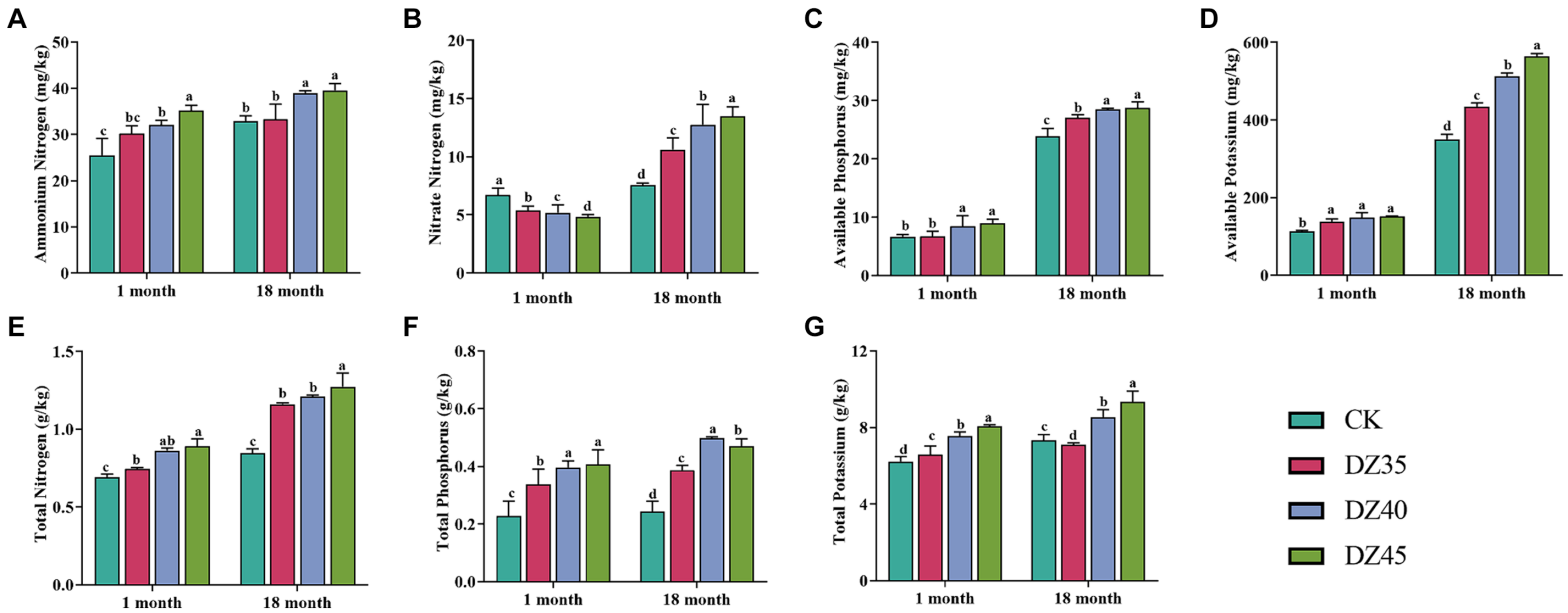
Effects of dazomet fumigation on the levels of ammonium nitrogen **(A)**, nitrate nitrogen **(B)**, available phosphorus **(C)**, available potassium **(D)**, total nitrogen **(E)**, total phosphorus **(F)** and total potassium **(G)** in soil. Lowercase letters indicate significance at *p* < 0.05 level (*n* = 3).

DZ fumigation could significantly promote the accumulation of nutrient elements in the soil. Compared to the CK, after 18 months of fumigation, the contents of TN, TP, TK, AK, AP, and NH_4_^+^-N in the soil were significantly increased after DZ fumigation, and the enhancement effect increased with the DZ application amount (Figure 3). One month after fumigation, compared to the CK, the content of NO_3_^−^-N in the soil significantly decreased, and a higher DZ concentration correlated with lower NO_3_^−^-N content. However, after 18 months of fumigation, DZ fumigation showed a significant promoting effect on the content of NO_3_^−^-N in the soil (Figure 3B). This indicated that over time, the inhibitory impact of DZ fumigation on soil nitrification gradually diminishes, ultimately facilitating soil nitrification.

**Figure 4:**
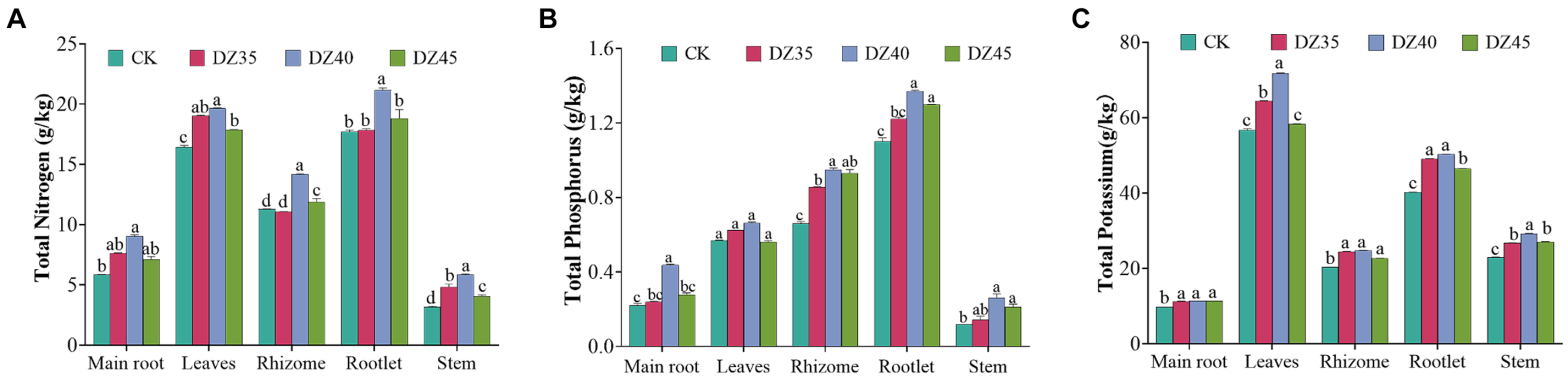
Effects of dazomet fumigation on the content of total nitrogen **(A)**, total phosphorus **(B)** and total potassium **(C)** of *P. notoginseng*. Lowercase letters indicate significance at *p* < 0.05 level (*n* = 3).

DZ fumigation promoted the accumulation of nitrogen, phosphorus, and potassium in *P. notoginseng* plants. After DZ fumigation, the content of nitrogen, phosphorus, and potassium in the main root, leave, rhizome, rootlet, and stems was significantly higher than that in the untreated control (CK), especially in the DZ40 treatment. The contents of nitrogen in the main root, leaves, rhizomes, rootlet, and stems increased by 54.36, 19.68, 25.62, 19.56, and 84.81% respectively, and the contents of phosphorus increased by 98.29, 16.28, 43.64, 24.52, and 122.43% respectively, and the contents of potassium increased by 15.91, 26.57, 21.45, 25.01, and 27.25,respectively, compared to the CK (Figure 4).

### 3.4 Effect of DZ fumigation on soil microorganisms

#### 3.4.1 Effect of DZ fumigation on the α-diversity of soil microbial communities

**Figure 5:**
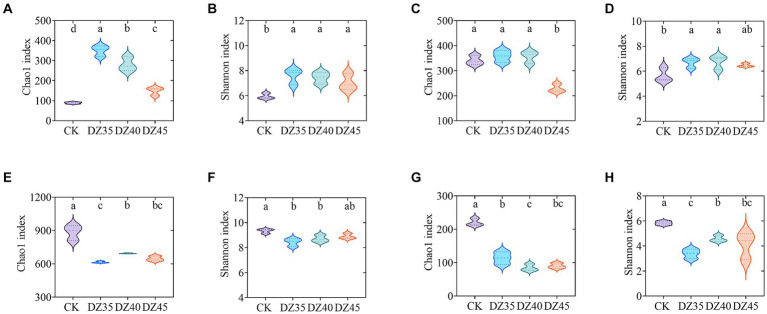
Alpha diversity indices of soil bacteria and fungi within dazomet fumigation. **(A)** The bacterial Chao1 index for one month; **(B)** The bacterial Shannon index for one month; **(C)** The fungal Chao1 index for one month; **(D)** The fungal Shannon index for one month; **(E)** The bacterial Chao1 index for 18 month; **(F)** The bacterial Shannon index for 18 month; **(G)** The fungal Chao1 index for18 month; **(H)** The fungal Shannon index for 18 month. Lowercase letters indicate significance at *p* < 0.05 level (*n* = 3).

At 1 month following DZ fumigation, the Chao1 and Shannon indices of soil bacteria were significantly increased compared to CK. A decrease was observed with the increase in DZ application amount (Figures 5A,B). DZ fumigation also resulted in increased Chao1 and Shannon indices of soil fungi, except for the DZ45 treatment, which showed a decrease with the increase in DZ concentration (Figures 5C,D).

At 18 months following DZ fumigation, the Chao1 and Shannon indices of bacteria and fungi in the soil were significantly lower than CK. The bacterial Chao1 and Shannon indices under DZ40 treatment were slightly higher than that of DZ35 and DZ40 (Figures 5E,F). The Chao1 index of fungi in the soil decreased overall with the increasing application amount of DZ, while the Shannon index had an overall increase with increasing application amount of DZ (Figures 5G,H).

#### 3.4.2 Effect of DZ fumigation on the composition of bacterial and fungal genera in soil

**Figure 6:**
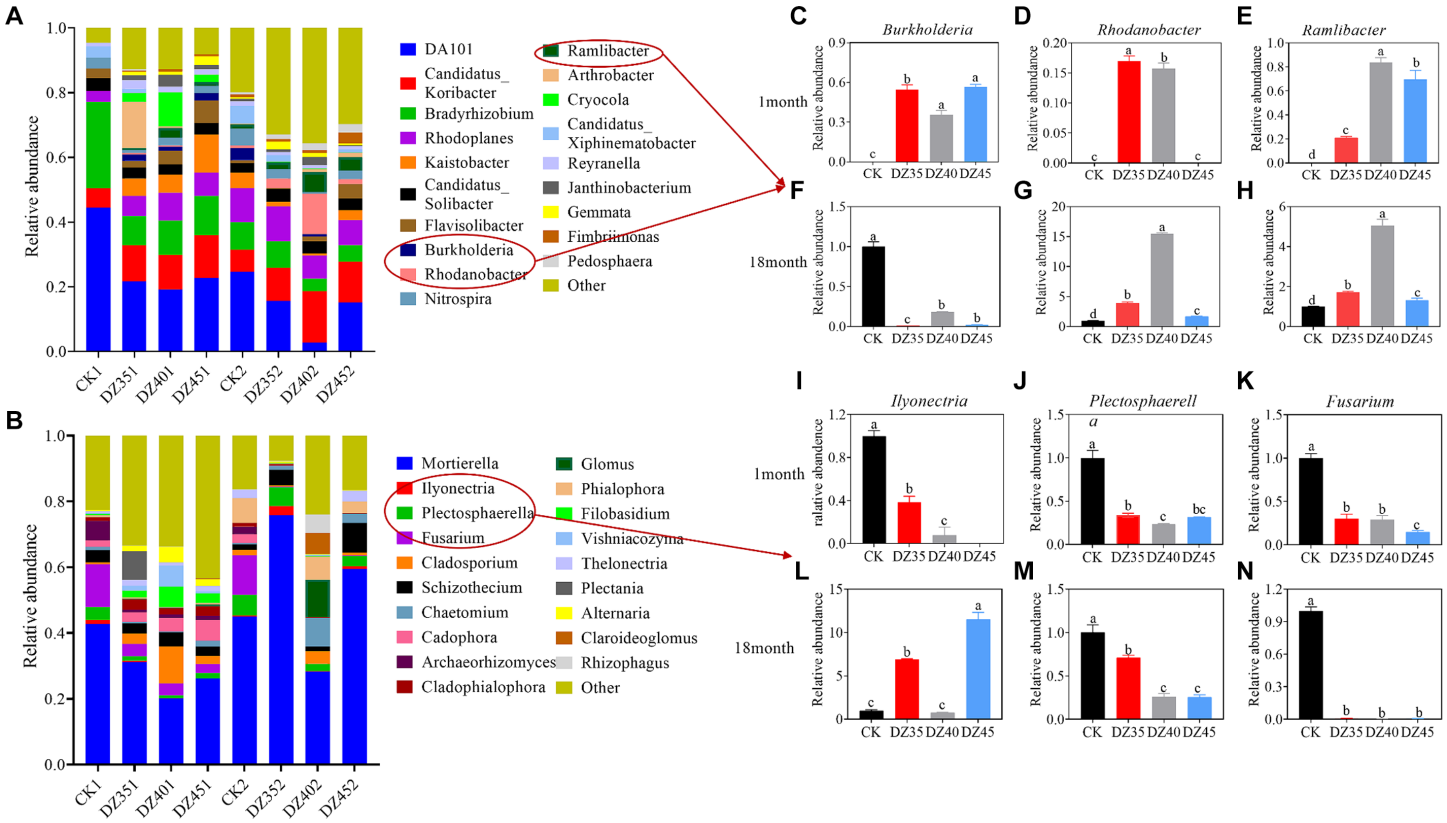
Effects of dazomet fumigation on the composition of soil bacteria **(A)** and fungi **(B)** at the genus level (TOP 20). The relative abundance of Burkholderia **(C)**, Rhodanobacter **(D)**, and Ramlibacter **(E)** for one month; The relative abundance of Burkholderia **(F)**, Rhodanobacter **(G)**, and Ramlibacter **(H)** for 18 month; The relative abundance of Ilyonectria **(I)**, Plectosphaerella **(J)**, and Fusarium **(K)** for one month; The relative abundance of Ilyonectria **(L)**, Plectosphaerella **(M)**, and Fusarium **(N)** for 18 month. Lowercase letters indicate significance at *p* < 0.05 level (*n* = 3).

The dominant bacterial genera in the soil included *DA101*, *Candidatus-Koribacter*, *Bradyrhizobium*, and *Rhodoplanes* (Figure 6A). DZ fumigation increased the relative abundance of *Candidatus-Koribacter* and *Rhodoplanes,* while decreasing the relative abundance of *DA101* and *Bradyrhizobium*. One month after fumigation, compared to CK, the relative abundance of *Candidatus-Koribacter* increased by 77.89, 63.86, and 77.55% with the DZ35, DZ40, and DZ45 treatments, respectively. The relative abundance of *Rhodoplanes* increased by 80.85, 137.89, and 74.67% in the same treatments. The relative abundance of *DA101* decreased by 53.99, 61.03, and 59.40%, and the relative abundance of *Bradyrhizobium* decreased by 68.44, 64.15, and 64.06%. DZ fumigation also promoted the growth of beneficial bacteria, such as *Rhodanobacter, Ramlibacter*, and *Burkholderia* (Figures 6C–E).

At 18 months after fumigation, compared to the CK, the relative abundance of *Candidatus-Koribacter* continued to increase, while the relative abundances of *DA101, Bradyrhizobium,* and *Rhodoplanes* showed decreasing trends. Specifically, compared to the CK, the relative abundance of *DA101* decreased by 4.16, 2.63, and 2.55% in the DZ35, DZ40, and DZ45 treatments, respectively. The relative abundance of *Bradyrhizobium* changed by −7.01, 50.44, and 43.01%, while that of *Rhodoplanes* changed by −11.65, 27.55, and 30.93%. In contrast, the relative abundance of *Candidatus-Koribacter* increased by 64.01, 150.91, and 74.85%, compared to CK (Figure 6A). The relative abundances of *Rhodanobacter* and *Ramlibacter* significantly increased compared to the CK, while the relative abundance of *Burkholderia* decreased by 89.66 to 99.60%. The highest relative abundance of *Rhodanobacter, Ramlibacter,* and *Burkholderia* were observed in the DZ40 treatment (Figures 6F–H).

*Mortierella* is a dominant soil fungus. DZ fumigation significantly reduced its relative abundance, and the relative abundance decreased with increasing DZ application. One month after fumigation, compared to CK, the relative abundance of *Mortierella* in the DZ35, DZ40, and DZ45 treatments decreased by 23.98, 51.13, and 56.43%, respectively (Figure 6B). DZ fumigation reduced the relative abundance of soil pathogens *Fusarium, Plectosphaerella* and *Ilyonectria*. Compared to CK, the relative abundance of *Fusarium* decreased by 70, 70.81, and 85.08% in the DZ35, DZ40, and DZ45 treatments, respectively. *Plectosphaerella* decreased by 66.49, 76.95, and 68.43% while *Ilyonectria* decreased by 61.44, 92.25, and 100% (Figures 6I–K).

At 18 months following fumigation, the relative abundance of *Mortierella* in the DZ35 treatment increased by 34.93% compared to the CK treatment, while the DZ40 and DZ45 treatments also showed suppressive effects on its relative abundance (Figure 6B). A strong inhibitory effects were still observed on the relative abundance of *Fusarium* and *Plectosphaerella* after DZ fumigation, with the relative abundance of *Fusarium* decreased by 99.03–99.66%, and the *Plectosphaerella* decreased by 28.29–74.36%, compared to the CK (Figures 6L–N).

#### 3.4.3 Correlation analysis of soil microbial communities

**Figure 7:**
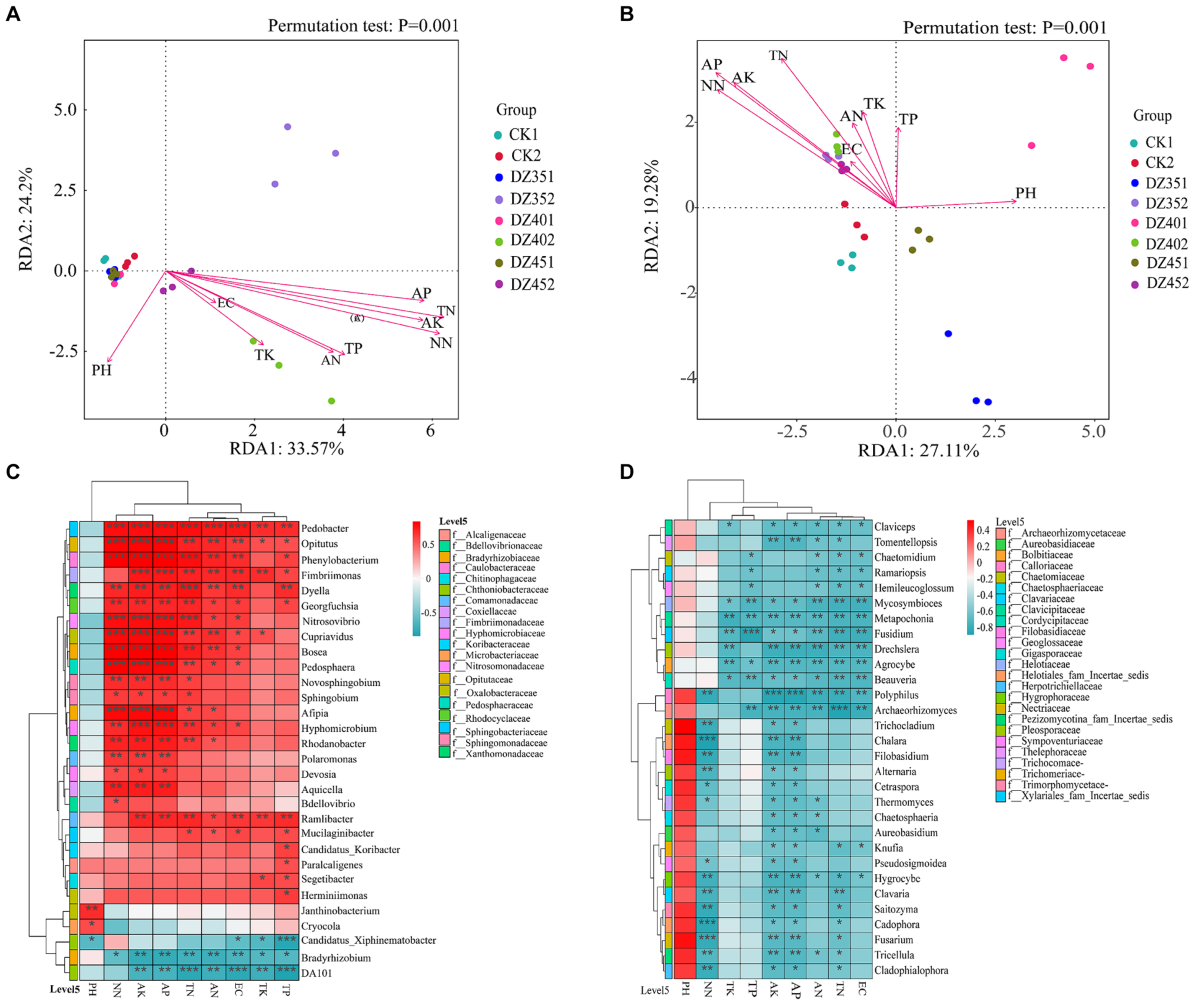
Redundancy analysis (RDA) and correlation heatmap analysis of soil physicochemical with microorganisms (**A**/**C**: bacteria, **B**/**D**: fungi).

In Figure 7A, the X and Y axes accounted for 33.57 and 24.2%, respectively, of the variation in the community structure at the bacterial genus level. Environmental factors such as TN, TP, AK, AP, AN, and NN had *p* values less than 0.01, indicating a significant correlation with the community structure. The pH and TK factors had *p* values of 0.015 and 0.016, respectively, which were considered statistically significant. However, EC, with a *p* value of 0.18, was not significant. This suggested that all environmental factors, except EC, significantly impacted the bacterial community structure. Among these factors, soil TN and NN content exhibited the strongest correlation, followed by AP, AK and TP. The correlation heatmap revealed a positive relationship between most bacterial genera and soil physicochemical properties, particularly with NN, AK, and AP. These findings were consistent with the results of the RDA analysis in Figure 7C. For instance, *Pedobacter* showed highly significant positive correlations with NN, AK, AP, TN, AN, and EC; *Opitutus, Phenylobacterium, Cupriavidus*, *Bosea, Pedosphaera*, and *Afipia* showed highly significant positive correlations with NN, AK, and AP; *Fimbriimonas* and *Hyphomicrobium* showed highly significant positive correlations with AP and AK; *Dyella* showed a highly significant positive correlation with TN; *Phenylobacterium* and *Nitrosovibrio* showed highly significant positive correlations with NN, AK, AP and TN; *Janthinobacterium* and *Cryocola* showed significant correlations with pH Conversely, *Bradyrhizobium* and DA101 had significant negative correlations with AK, AP, TN, AN, EC, TK and TP.

In Figure 7B, the X and Y axes accounted for 27.11 and 19.28%, respectively, of the variation in the horizontal community structure of fungi. Except for TP and EC, other environmental factors significantly explained this variation, with pH, TN, AP, AK and NN having *p* values less than 0.01, indicating a highly significant relationship. Specifically, AP content in the soil showed the strongest correlation with fungal community structure, followed by NN, AK and TN. The correlation heatmap analysis in Figure 7D demonstrated that most fungal genera have negative correlations with soil physicochemical indicators, except for pH. Notably, *Chalara, Cadophora* sp., and *Fusarium* showed significant negative correlations with NN; *Fusidium* had a negative correlation with TP; *Polyphilus* exhibited positive correlations with AK and AP; and *Archaeorhizomyces* showed a negative correlation with TN.

#### 3.4.4 Functional prediction of soil microorganisms

**Figure 8:**
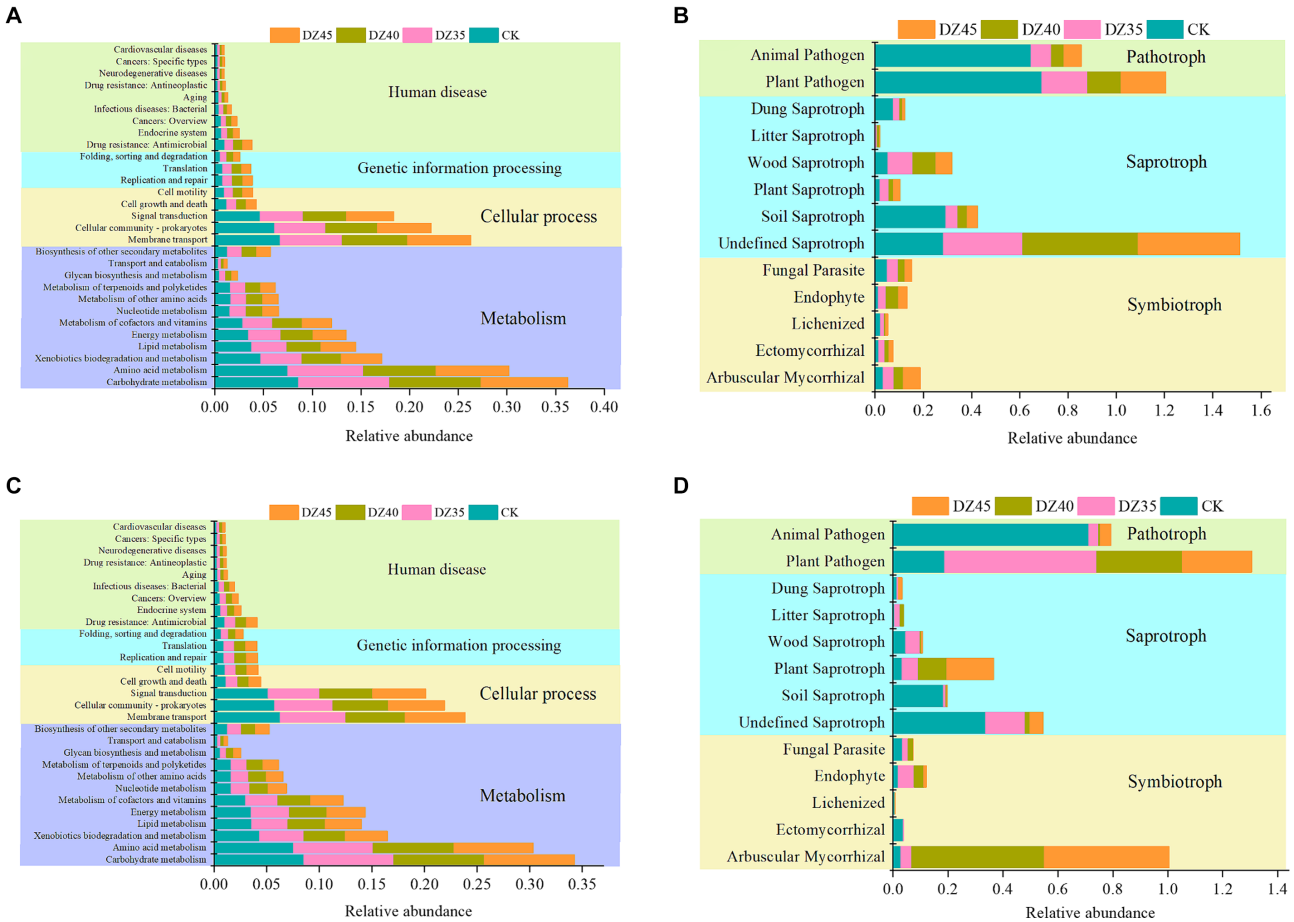
Prediction analysis of soil microorganisms at 1 month (**A**: bacteria, **B**: fungi) and 18 months after fumigation (**C**: bacteria, **D**: fungi).

After fumigation, soil bacterial functions in Level 1 of the KEGG database were primarily categorized into metabolism, cellular processes, genetic information processing, and human diseases. Metabolism-related pathways constituted 59.09 to 59.12%, while cellular processes made up 30.44 to 30.78%, with the lowest percentage associated with human diseases and genetic information processing (Figure 8). At 1 month after fumigation, there was an increase in the relative abundance of certain functional bacterial communities participating in metabolism and genetic information processing. However, the majority of functional bacterial communities declined in relative abundance. Compared to CK, bacterial groups linked to carbohydrate metabolism, cofactor and vitamin metabolism, nucleotide metabolism, biosynthesis of other secondary metabolites, amino acid metabolism, replication and repair, and translation showed increases, with DZ40 treatment displaying the highest relative abundance (Figure 8A). At 18 months after fumigation, bacterial groups associated with amino acid metabolism, cofactor and vitamin metabolism, nucleotide metabolism, other amino acids metabolism, transport and catabolism, and biosynthesis of other secondary metabolites showed a higher relative abundance in the DZ40 treatment compared to CK, DZ35, and DZ45 treatments (Figure 8C). Bacterial groups involved in biosynthesis of other secondary metabolites exhibited higher relative abundance compared to other treatments, correlating with the higher PNS content of secondary metabolites in the DZ40 treatment compared to other treatments.

Soil fungi can be categorized, based on their nutrition modes, into pathotroph pathogen, symbiotroph pathogen, and saprotroph pathogen. In this study, the fungal communities in fumigated soil revealed shifts in their functional composition because of DZ fumigation. One month after fumigation, the relative abundances of arbuscular mycorrhizal-ectomycorrhizal fungi and undefined-plant-wood-litter saprotrophic fungi increased, while the relative abundance of plant–animal pathogen fungi significantly decreased. In comparison to the CK, the relative abundances in the DZ35, DZ40, and DZ45 treatments declined by 79.59, 85.78, and 80.65%, respectively, (Figure 8B). This indicated that DZ fumigation enhanced the proliferation of soil saprotrophic fungi involved in decomposing plant residues, promoting carbon and nitrogen cycling and concurrently reducing the relative abundance of pathogenic fungi. These changes reduced the risk of infection and incidence of soil-borne disease.

At 18 months after fumigation, the relative abundance of arbuscular mycorrhizal-ectomycorrhizal fungi in the soil remained higher than that in the CK treatment. The relative abundance of these fungi in the DZ40 and DZ45 treatments was 642.92 and 599.32% higher than in the CK treatment, respectively. Conversely, the relative abundances of saprotrophic fungi and plant–animal pathogenic fungi were lower than those in the CK treatment. With an increasing DZ application amount, the relative abundance of plant–animal pathogenic fungi in the soil decreased (Figure 8D).

## 4 Discussion

Soil microbes regulate soil ecology and soil immunity, which are critical for maintaining soil health. Significant negative plant–soil feedback (NPSF) exists between *P. notoginseng* and soil. Throughout *P. notoginseng* growth, the accumulation of root exudates in the rhizosphere soil can inhibit the growth of beneficial bacteria and facilitate the proliferation of pathogenic fungi. This leads to an imbalance in rhizosphere microbial ecology, which is the main driver of *P. notoginseng-soil* NPSF (Wei et al., 2018; Luo et al., 2019). Fumigation can alter the ratio of pathogenic and beneficial bacteria and reshape the ecological balance of soil microorganisms (Ibekwe et al., 2001). *Fusarium, Plectosphaerella* and *Ilyonectria* are pathogens that cause plant wilt, root rot, and rust diseases. These species pose challenges for consecutive cropping of tomato and *P. notoginseng* (Gao et al., 2021; Luo et al., 2022). The present study revealed that DZ fumigation significantly inhibited the growth of *Fusarium, Plectosphaerella*, and *Ilyonectria* in the test soil, with the inhibitory effect persisting for up to 18 months after fumigation. DZ fumigation also increased the relative abundance of beneficial bacteria, including *Ramlibacter, Rhodanobacter* and *Burkholderia* in the soil, with the levels of *Ramlibacter* and *Rhodanobacter* remaining significantly elevated 18 months after fumigation (Figure 6). The main reason for this phenomenon is that the degradation product of DZ in soil (methyl isothiocyanate) can kill *Fusarium* by interfering with its reactive oxygen species scavenging capacity (Zhang et al., 2024), and *Rhodanobacter* has an antagonistic effect on Fusarium, which can effectively inhibit the growth of Fusarium (Huo et al., 2018). *Burkholderia* as a soil biocontrol bacterium, which can alleviate the NPSF and improve the emergence rate and fresh weight of *P. notoginseng* plants (Santos et al., 2004; Elshafie et al., 2012; Esmaeel et al., 2019; Luo et al., 2019). *Ramlibacter* and *Rhodanobacter* played significant roles in the soil nitrogen cycle, contributing to the enhancement of soil fertility (Yu et al., 2019). Overall, DZ fumigation improved the soil microecological environment, alleviated the NPSF of *P. notoginseng* and promoted its growth and development.

The soil environment, encompassing pH, N, P, K, temperature, and humidity, is crucial in shaping the structure and diversity of soil microbial communities. Strong associations exist between specific microbial groups and soil variables (Hermans et al., 2020). Our results showed that soil AP, AN, NN were significantly associated with the taxonomic structure of fungal genera and the pathogenic fungus *Fusarium* exhibited a highly significant negative correlation with soil NN (Figure 7B). An increase in NN content in the soil effectively regulated the incidence of root rot disease in *P. notoginseng*, consistent with previous research (Ren et al., 2021; Zhao et al., 2023). These data suggest that managing NN levels can help control *P. notoginseng* diseases induced by *Fusarium* and other fungi positively correlated with *Fusarium*. This management could reduce pesticide usage and promote sustainable cultivation of *P. notoginseng*.

The nutritional environment in the soil is critical in influencing the distribution and composition of soil microbiota, which, in turn, drives soil nutrient cycling. Soil microbiota are involved in nutrient transformation and regulation of nutrient types and quantities. DZ fumigation can modify the abundance and community structure of denitrifying bacteria, suppress nitrification, and promote the conversion of nitrate into ammonium nitrogen (Fang et al., 2020; Sennett et al., 2022). In the present study, the TN and NH_4_^+^-N contents in the soil after DZ fumigation were significantly elevated compared to CK (Figures 3A,B,E). This increase may be attributed to the increased abundance of *Rhodanobacter* and *Ramlibacter* in the soil following DZ fumigation, which enhance denitrification and nitrate assimilation reduction processes and increase NH_4_^+^-N content (Lee et al., 2007; Prakash et al., 2012; Huang et al., 2021; Hu et al., 2023).

Soil microbial activity is the primary force driving phosphorus transformation and cycling. DZ fumigation decreases the abundance and diversity of soil microorganisms carrying *phoD*, promotes phosphorus mineralization and results in a rapid increase of AP content (Huang et al., 2020). We also found that DZ fumigation augmented the levels of AP and TP in the soil (Figures 3C,F). The release of phosphorus-containing substances such as nucleic acids post-microbial death resulting from fumigation, combined with the increased relative abundance of phosphate-solubilizing bacteria like *Burkholderia* due to DZ fumigation, facilitates the dissolution of insoluble phosphates. This, consequently, elevates soil phosphorus content (Lin et al., 2006; Weisskopf et al., 2011).

Despite previous reports concluding that DZ fumigation did not significantly affect soil potassium content and even decreased the AK level (Li et al., 2017, 2023; Chen et al., 2022), we observed an increase in AK and TK levels in the soil after DZ fumigation (Figures 3D,G). This rise may be attributed to the enhanced relative abundance of potassium-solubilizing microbes (KSM) such as *Burkholderia* and *Arthobacter* as a result of DZ fumigation, which promoted the release and dissolution of mineral potassium (Sharma et al., 2024). Additionally, DZ fumigation led to an upsurge in the relative abundance of saprophytic fungi, which accelerated plant residue decomposition, enhanced potassium release from plants into the soil, thereby increasing soil potassium content.

Soil serves as the medium for plant growth, with soil microbial diversity crucial for maintaining plant productivity. Plant nutrient uptake and stress resistance rely heavily on soil microbes (Chen et al., 2020). Compared to the high metabolic consumption and absorption of root systems, plants typically acquire nutrients from the soil environment through fungal networks (Felderer et al., 2013). Arbuscular mycorrhizal fungi (AMF), as obligate symbiotic organisms engaging in root symbiosis, establish a mutualistic association with plant roots (Gadkar et al., 2001). AMF can enhance plant water and nutrient absorption capabilities (P and N) by improving physiological morphology and thereby promote plant growth (Ruiz-Lozano et al., 2016; Andrino et al., 2021; Jing et al., 2022). In addition, arbuscular mycorrhizal fungi can also increase the medicinal active ingredients of medicinal plants by enhancing biomass or secondary metabolic synthesis pathways. The promoting effects are most significant for flavonoids (68%) and terpenes (53%), and there is a significant increase in the content of medicinal components in underground parts (Zeng et al., 2013; Zhao et al., 2022; Ren et al., 2023; Yuan et al., 2023). We speculate that the fumigation with DZ increased the relative abundance of arbuscular mycorrhizal fungi in the soil, increased the infection rate of AMF in Sanqi, and ultimately led to a significant increase in biomass and saponin content of P. notoginseng (Figures 2, 4; Supplementary Table 1; Supplementary Figure 1).

Soil fumigation has been practiced in China for almost 70 years. While it helps to decrease pesticide and fertilizer usage and enhance crop yield, the continuous application of DZ can hasten its breakdown, diminishing its efficacy in managing soil-borne diseases (Di Primo et al., 2003). In regions where DZ is frequently applied, farmers may opt to escalate the dosage for improved fumigation outcomes. Nevertheless, DZ fumigation can elevate the total abundance and diversity of antibiotic resistance genes (ARGs) in the soil, with this rise positively associated with the dazomet dosage (Zhang et al., 2023). The soil–plant system is a significant pathway for the acquisition and dissemination of ARGs. Further research is needed to determine if DZ fumigation will elevate the risk of human exposure to ARGs through the *P. notoginseng* chain.

## 5 Conclusion

This study found that DZ fumigation before planting *P. notoginseng* reshaped the ecological balance of soil microbiota by reducing the relative abundance of pathogenic fungi such as *Fusarium, Plectosphaerella* and *Ilyonectria*, while increasing the relative abundance of beneficial bacteria like *Ramlibacter, Burkholderia* and *Rhodanobacters*. This restructuring promoted the favorable development of soil microecology, leading to effective control of soil-borne diseases and enhancing the emergence and survival rates of *P. notoginseng*. DZ fumigation improved the soil microbial environment and increased levels of TN, TP, AP, and NH_4_^+^-N. These modifications promoted the absorption and utilization of nutrients, as well as the growth and development of *P. notoginseng*. Consequently, these changes resulted in increased saponin accumulation, thereby improving the quality of *P. notoginseng*. In conclusion, DZ fumigation effectively addresses the challenges of continuous cropping with *P. notoginseng*, enhancing both its yield and quality.

## Data availability statement

The datasets presented in this study can be found in online repositories. The names of the repository/repositories and accession number(s) can be found in the article/Supplementary material.

## Author contributions

Y-mL: Conceptualization, Data curation, Formal analysis, Methodology, Project administration, Supervision, Validation, Writing – original draft, Writing – review & editing. M-hL: Data curation, Formal analysis, Methodology, Project administration, Validation, Writing – original draft. C-yD: Supervision, Visualization, Writing – review & editing. YL: Methodology, Visualization, Writing – review & editing. W-pZ: Investigation, Methodology, Writing – review & editing. QY: Conceptualization, Methodology, Writing – review & editing. X-mC: Resources, Writing – review & editing. YY: Conceptualization, Data curation, Funding acquisition, Resources, Writing – review & editing.

## Footnotes

^1^ https://international.biocloud.net/
