# Supplementary material for: Dazomet fumigation modification of the soil microorganism community and promotion of Panax notoginseng growth
Source: Front Microbiol. 2024 Jul 26;15:1443526. doi: 10.3389/fmicb.2024.1443526 (PMC11309993; doi:10.3389/fmicb.2024.1443526)
Supplement: Supplementary file 1 [file Data_Sheet_1.docx]

Supplementary Figures and Tables

1 Supplementary Figures

Supplementary Figure1. Effects of different concentrations of dazomet fumigation on the agronomic traits of *P. notoginseng*. Lowercase letters indicate significance at the *P* < 0.05 level (n = 15).

2 Supplementary Tables

Supplementary Table 1. Effects of different treatment on the growth of *P. notoginseng* leaves（n=15）

| Treatment | Leaf width（mm） | Leaf length（mm） | leaf area  （mm^2^） | Length-width ratio | Leaf weight（g） |
| --- | --- | --- | --- | --- | --- |
| CK | 37.84±5.43a | 43.80±9.72c | 1387.43±195.89b | 1.16±0.25b | 3.12±0.86b |
| DZ35 | 38.26± 5.74a | 47.87±8.83bc | 1415.56±189.89b | 1.24±0.26b | 3.65±0.64ab |
| DZ40 | 39.66±4.97a | 50.53±9.75ab | 1570.73±180.89ab | 1.30±0.21ab | 3.96±0.72a |
| DZ45 | 39.42±5.86a | 48.67±9.94bc | 1473.44±297.33ab | 1.29±0.18ab | 3.69±0.79ab |

Supplementary Table 2. Effects of dazomet fumigation on the genus Top 20 of bacteria in soil（n=3）

|  | 1 month | | | | 18 month | | | |
| --- | --- | --- | --- | --- | --- | --- | --- | --- |
| Genus | CK1 | DZ351 | DZ401 | DZ451 | CK2 | DZ352 | DZ402 | DZ452 |
| *DA101* | 0.138946 | 0.063936 | 0.054145 | 0.056415 | 0.07517 | 0.05229 | 0.009051 | 0.043591 |
| *Candidatus_Koribacter* | 0.018486 | 0.0328852 | 0.030292 | 0.032822 | 0.020591 | 0.03377 | 0.051664 | 0.036003 |
| *Bradyrhizobium* | 0.083549 | 0.0263681 | 0.02995 | 0.030024 | 0.025932 | 0.027772 | 0.012851 | 0.014778 |
| *Rhodoplanes* | 0.010193 | 0.0184342 | 0.024248 | 0.017804 | 0.03196 | 0.035685 | 0.023156 | 0.022074 |
| *Candidatus_Solibacter* | 0.013014 | 0.0099421 | 0.008999 | 0.008887 | 0.00906 | 0.013215 | 0.012481 | 0.01031 |
| *Kaistobacter* | 0 | 0.0160507 | 0.01579 | 0.029267 | 0.014633 | 0.004518 | 0.001994 | 0.008778 |
| *Flavisolibacter* | 0.009198 | 0.0061467 | 0.011495 | 0.017063 | 0.002721 | 0.000731 | 0.004772 | 0.012615 |
| *Burkholderia* | 0 | 0.005488 | 0.003564 | 0.005658 | 0.011258 | 0.000118 | 0.002055 | 0.00023 |
| *Rhodanobacter* | 0 | 0.001693 | 0.001561 | 0 | 0.00265 | 0.01033 | 0.041005 | 0.004492 |
| *Nitrospira* | 0.010045 | 0.0022899 | 0.006545 | 0.005572 | 0.015673 | 0.009336 | 0.001421 | 0.007541 |
| *Ramlibacter* | 0 | 0.0020663 | 0.00837 | 0.002768 | 0.003977 | 0.006846 | 0.02009 | 0.011586 |
| *Arthrobacter* | 0 | 0.0421369 | 0.001547 | 0 | 0.000541 | 0.00035 | 0.003117 | 0.00403 |
| *Cryocola* | 0 | 0.0081199 | 0.029394 | 0.005938 | 0 | 0.000828 | 0.000544 | 0.000178 |
| *Candidatus_Xiphinematobacter* | 0.011058 | 0.0036682 | 0.00026 | 0 | 0.016542 | 0.006408 | 0 | 0.002732 |
| *Reyranella* | 0.003525 | 0.0077437 | 0.004991 | 0.003969 | 0.00458 | 0.003225 | 0.00353 | 0.003135 |
| *Janthinobacterium* | 0 | 0.0046093 | 0.010042 | 0.002992 | 0.002455 | 0.002749 | 0.007932 | 0.000447 |
| *Gemmata* | 0 | 0.0032294 | 0.002995 | 0.007164 | 0.001373 | 0.007901 | 0.00422 | 0.00186 |
| *Fimbriimonas* | 0 | 0.0010031 | 0.001921 | 0.001591 | 0.002557 | 0.002982 | 0.002772 | 0.009906 |
| *Pedosphaera* | 0 | 0.0009403 | 0 | 0 | 0.001719 | 0.004412 | 0.006858 | 0.007183 |
| *Others* | 0.014281 | 0.0378546 | 0.036094 | 0.019974 | 0.060923 | 0.109775 | 0.116125 | 0.085109 |
|  |  |  |  |  |  |  |  |  |

Supplementary Table 3. Effects of dazomet fumi gation on the genus Top 20 of fungi in soil（n=3）

|  | 1 month | | | | | 18 month | | | | | | |
| --- | --- | --- | --- | --- | --- | --- | --- | --- | --- | --- | --- | --- |
| Genus | CK1 | DZ351 | | DZ401 | DZ451 | CK2 | DZ352 | | DZ402 | | DZ452 | |
| *Mortierella* | 0.272905 | 0.207467 | | 0.133363 | 0.118914 | 0.32546 | 0.439136 | | 0.239551 | | 0.222364 | |
| *Ilyonectria* | 0.00861 | 0.00332 | | 0.000667 | 0 | 0.002326 | 0.0161 | | 0.001712 | | 0.026728 | |
| *Plectosphaerella* | 0.02521 | 0.008447 | | 0.00581 | 0.007958 | 0.04569 | 0.03249 | | 0.017678 | | 0.011717 | |
| *Schizothecium* | 0.023787 | 0.020922 | | 0.027822 | 0.012934 | 0.012387 | 0.027773 | | 0.012381 | | 0.032773 | |
| *Cladosporium* | 0.004111 | 0.020964 | | 0.075124 | 0.011149 | 0.012067 | 0.002564 | | 0.0311 | | 0.003093 | |
| *Chaetomium* | 0.006555 | 0.002918 | | 0.002196 | 0.007983 | 0.003161 | 0.007071 | | 0.070262 | | 0.009902 | |
| *Cadophora* | 0.012436 | 0.019514 | | 0.027383 | 0.028983 | 0.018978 | 0.000525 | | 0 | | 0.000492 | |
| *Glomus* | 0.000768 | 0.001383 | | 0.001239 | 0.002616 | 0.000444 | 0 | | 0.092575 | | 0 | |
| *Phialophora* | 0.003227 | 0.002291 | | 0.001375 | 0.002186 | 0.05383 | 0.001152 | | 0.058113 | | 0.012652 | |
| *Fusarium* | 0.08196 | 0.024587 | | 0.023926 | 0.01223 | 0.08638 | 0.000839 | | 0.000295 | | 0.000653 | |
| *Archaeorhizomyces* | 0.038077 | 0.005975 | | 0.006872 | 0.005439 | 0.016672 | 0 | | 0.000767 | | 0.000049 | |
| *Filobasidium* | 0.002558 | 0.013827 | | 0.041582 | 0.012493 | 0 | 0.000758 | | 0.000409 | | 0 | |
| *Cladophialophora* | 0.006509 | 0.020688 | | 0.011878 | 0.013387 | 0.007788 | 0.00247 | | 0.001355 | | 0.000352 | |
| *Vishniacozyma* | 0.001514 | 0.009854 | | 0.042937 | 0.002729 | 0 | 0.000211 | | 0.004907 | | 0 | |
| *Thelonectria* | 0.004605 | 0.011371 | | 0.005888 | 0.007955 | 0.018538 | 0 | | 0 | | 0.012474 | |
| *Plectania* | 0 | 0.058442 | | 0 | 0 | 0 | 0 | | 0 | | 0 | |
| *Alternaria* | 0.002407 | 0.010847 | 0.031486 | | 0.009608 | 0.000157 | 0.002626 | 0.00046 | | 0.000812 | |  |
| *Claroideoglomus* | 0.000055 | 0.000343 | 0.000289 | | 0.000426 | 0 | 0 | 0.052426 | | 0 | |  |
| *Rhizophagus* | 0 | 0.000202 | 0.000466 | | 0.000376 | 0.00066 | 0.000968 | 0.045677 | | 0.000075 | |  |
| *Other* | 0.144009 | 0.222545 | 0.223918 | | 0.197648 | 0.11847 | 0.044349 | 0.196185 | | 0.060242 | |  |
